# Targeting the *ZMYM2–ANXA9* Axis: Organoid Models Illuminate a Novel Pathway to Overcome Oxaliplatin Resistance in Colorectal Cancer

**DOI:** 10.3390/biomedicines13112804

**Published:** 2025-11-18

**Authors:** Shi-Jie Zeng, Ke He, Zhi Shi

**Affiliations:** Cancer Minimally Invasive Therapies Centre, Guangdong Second Provincial General Hospital, Department of Cell Biology & Institute of Biomedicine, Guangdong Provincial Biotechnology & Engineering Technology Research Center, Guangdong Provincial Key Laboratory of Bioengineering Medicine, Guangdong Basic Research Center of Excellence for Natural Bioactive Molecules and Discovery of Innovative Drugs, Genomic Medicine Engineering Research Center of Ministry of Education, MOE Key Laboratory of Tumor Molecular Biology, National Engineering Research Center of Genetic Medicine, State Key Laboratory of Bioactive Molecules and Druggability Assessment, College of Life Science and Technology, Jinan University, Guangzhou 510632, China; zengshijie@stu2024.jnu.edu.cn (S.-J.Z.); heke8@mail3.sysu.edu.cn (K.H.)

Colorectal cancer (CRC) represents a major public health challenge. The 2022 Global Cancer Statistics Report revealed that CRC accounted for over 1.9 million new cases and more than 900,000 deaths globally, ranking as the third most frequently diagnosed cancer and the second leading cause of cancer mortality [1]. Notably, China is experiencing a rapidly increasing burden of CRC, with new cases projected to rise from 560,000 in 2020 to 910,000 by 2040, posing a formidable challenge to the country’s cancer prevention and treatment systems [2]. Inherited genetic syndromes significantly increase individual susceptibility to CRC [3,4,5], while poor diet and unhealthy lifestyle have been identified as major contributing factors [6,7,8,9]. Due to the subtle and often nonspecific early symptoms, the majority of patients are diagnosed at an advanced stage, when treatment options are considerably limited. Therefore, promoting early screening and reinforcing preventive strategies are crucial for reducing CRC mortality.

The clinical management of CRC involves a multimodal approach, including surgery, radiotherapy, chemotherapy, and immunotherapy [10,11,12,13,14,15]. For early-stage disease, curative treatment primarily relies on surgical resection, whereas systemic chemotherapy forms the cornerstone of management for advanced-stage patients and is often combined with other modalities. Platinum-based agents such as oxaliplatin have demonstrated clear efficacy in CRC treatment, both as monotherapy and in combination regimens such as folinic acid, fluorouracil, and oxaliplatin [16,17,18].

However, the development of resistance to oxaliplatin represents a major clinical obstacle, arising from a multifactorial process driven by diverse molecular mechanisms [19,20]. For instance, the activity of efflux transporters like multidrug resistance protein 2 has been linked to reduced intracellular accumulation of oxaliplatin in CRC models, thereby diminishing its cytotoxic effects [21,22]. At the cellular stress and deoxyribonucleic acid (DNA) repair, proteins such as xeroderma pigmentosum group A binding protein 2 promote the DNA damage response, mitigating oxaliplatin-induced DNA damage and enhancing resistance [23,24,25]. Evidence also confirms that aldo-keto reductase family 1 member C1 plays a critical role in regulating oxaliplatin resistance in CRC [26]. Small-molecule inhibitors targeting aberrant cell-cycle pathways, such as polo-like kinase 1 inhibitors, can enhance the anti-tumor effect of oxaliplatin in CRC [27]. Moreover, the sensitivity of CRC cells to oxaliplatin is affected by certain non-coding ribonucleic acids (RNAs) [28,29]. In this context, exploring novel molecular mechanisms and therapeutic targets is crucial to overcoming chemotherapy resistance and improving patient prognosis.

The annexin family, a group of calcium-dependent phospholipid-binding proteins, participates in various cellular processes, including membrane transport, proliferation, apoptosis, and inflammation. Among them, the role of Annexin A9 (*ANXA9*) in CRC is increasingly being elucidated. Both mRNA and protein levels of *ANXA9* in CRC tissues are significantly higher compared to adjacent normal tissues, and its expression is closely correlated with tumor-node-metastasis stage and poor prognosis [30,31]. *ANXA9* promotes the proliferation, migration, and invasion of CRC cells and is involved in modulating anti-tumor immunity [32,33]. Together, these results indicate that *ANXA9* has potential as both a prognostic biomarker and a promising therapeutic target.

A pivotal study by Lin et al. in *Biomedicines* provides crucial insights into this issue by elucidating the role of *ANXA9* in oxaliplatin resistance. Integrating bioinformatics analysis with clinical sample validation, the authors systematically demonstrated for the first time that *ANXA9* mediates oxaliplatin resistance in CRC. They not only confirmed significant *ANXA9* overexpression in oxaliplatin-resistant cell lines and chemotherapy-insensitive patient tissues but also showed through functional experiments that *ANXA9* knockdown markedly enhanced oxaliplatin sensitivity, thereby inhibiting tumor cell proliferation and colony formation. Mechanistic studies revealed that zinc finger MYM-type containing 2 (*ZMYM2*) directly binds to the *ANXA9* promoter, with significantly stronger binding affinity in oxaliplatin-resistant cells, thereby enhancing transcription [34]. This work delineates the mechanism underlying *ANXA9* overexpression in resistant cells and identifies the novel *ZMYM2–ANXA9* signaling axis, providing fundamental insights into oxaliplatin resistance in CRC.

A key innovation of this study lies in its use of CRC patient-derived organoids as a disease model. Organoids offer a superior platform compared to traditional systems by closely recapitulating the 3D architecture, genetic profile, and heterogeneity of in vivo tumors, making them particularly valuable for drug sensitivity prediction [35,36,37]. High-throughput drug screening using this platform revealed that the fms-like tyrosine kinase 3 (*FLT3*) inhibitor G749 significantly enhances oxaliplatin sensitivity in organoids by downregulating *ANXA9* expression in a concentration-dependent manner. This finding not only validates the feasibility of targeting the *ZMYM2*–*ANXA9* axis but also underscores the value of organoid models in bridging basic research and clinical translation.

In summary, the identification of the *ZMYM2–ANXA9* axis unveils a pivotal mechanism underlying oxaliplatin resistance in CRC. By leveraging organoid models, the authors further demonstrate that the *FLT3* inhibitor G749 targets this axis and reverses resistance in preclinical settings. This work not only deepens our understanding of the oxaliplatin resistance mechanism in CRC but also identifies a promising candidate drug to overcome oxaliplatin resistance. Therefore, elucidating the therapeutic mechanism and comprehensive safety profile of G749 will be crucial to accelerate its translation into clinical trials, with the ultimate goal of addressing a pressing unmet need in oxaliplatin-resistant CRC.
